# Valedictory Address

**Published:** 1853-03

**Authors:** N. S. Davis

**Affiliations:** Professor of Pathology, Practice of Medicine, and Clinical Medicine


THE NORTH-WESTERN

MEDICAL AND SURGICAL JOURNAL.

NEW SERIES.

VOL. I. MARCH, 1853. No. 11.

### ORIGINAL COMMUNICATIONS.

ART. I.—*Valedictory Address to the Graduating Class in Rush Medical College.* By N. S. Davis, M.D., *Professor of Pathology, Practice of Medicine, and Clinical Medicine.*

Graduates and Gentlemen:

The present occasion marks the close of another annual term of instruction in Rush Medical College. The toils, the anxieties, the hopes and fears, the daily comminglings, and the opportunities for improvement, that accompanied it, are all now numbered with the things of the past. And owing to that sudden bereavement which has left a vacant place around the fireside of one of my most worthy colleagues, I am unexpectedly called upon to extend to you the customary congratulations, and formally recognize you as *Brethren* in the time-honored and noble profession of Medicine.

When the weather-beaten mariner has toiled through a season of storms, with the swelling waves of old ocean beneath, and the heavens veiled with clouds and darkness above, and finds himself at length in a calm but untried sea, with the king of day just throwing his silvery rays in dazzling beauty over the boundless expanse, his first object is to take his soundings, ascertain his latitude and longitude, and note the pointing of the needle, for the purpose of determining whither he is bound and the dangers that may beset his pathway. If the period of medical pupilage cannot be justly styled one of storms, it may, at least, be called an eventful portion of the voyage of life. It has its periods of sunshine and pleasant breezes, when the intellect is rapidly possessing itself of the choicest flowers of literature and the most beneficent truths of Science. It has its period of clouds and darkness, when the mind vainly endeavors to grasp the obscure, the intricate, and the unknown. It has its season, too, of storms, when the mind, weighed down with a sense of approaching responsibility and tortured with anxiety concerning the question of success or failure in regard to the attainment of the more immediate object of the student's ambition, namely, the formal and honorable admission into the ranks of a learned profession.

It is from this latter season, gentlemen, that you have just emerged; and now, while you are standing calmly, perhaps buoyantly, on the borders of what is to most of you a new and untried field, I can think of nothing more appropriate, than, like the mariner, to take the lead and line, the compass, and the Quadrant, and briefly explore the sea before you. The present aspect of Medical Science corresponds closely with that presented by any one or all the other sciences. A relationship which has been equally observable at every period since the days of the renowned sage of Cos.

When a crude system of Astrology was almost the only representative of natural science, diseases and medicines were alike supposed to be under the control of the stars.

When Fire, Earth, Air, and Water, were supposed to be the primary elements of the Universe, and all physical changes in matter were the results of concoction and fermentation, then Physiology consisted in a supposed knowledge of four Humors, called Blood, Phlegm, Bile, and Atrabile—and morbid action or disease consisted in excess, deficiency, or perversion of these.

When the Alchemists were laying the foundation of modern chemistry by their search after the Philosopher's-stone that should convert all the baser metals into Gold, Paracelsus and his followers were equally earnest in their search after the grand Panacea, the Elixir of Life, that was to rob the grim messenger of his prey and render the present life immortal. In more modern times, a still closer relationship is discernible between the progress of the different branches of Medical science and the natural sciences, technically so called.

So close, indeed, has been this relationship that scarcely a step of advancement has been taken in either of the latter, without having been productive, either directly or indirectly, of an equal advance in the former. As illustrations of this, we need only refer to the discoveries in the physiology of the nervous system by Sir Charles Bell, and their intimate connection with the cotemporaneous cultivation of natural history and comparative Anatomy — to that beautiful application of a knowledge of Acoustics or the laws of the transmission of sound to the more accurate appreciation of diseases of the heart, lungs, &c. — to that ingenious combination of mechanical principles found in the Surgical Adjuster — to that still more ingenious and valuable philosophical instrument, the compound Achromatic Microscope, which, by its applications in the study of minute, and pathological anatomy, has revealed to us a new and fascinating world of objects, and brought within our view the ultimate cells, fibres, and granules of which the human frame, with all its complex and wonderful combinations, is made — and last, though not least, we are reminded, at the bed-side of the sick, by each of those neat and concentrated therapeutic agents fresh and pure from the laboratory, of the inseparable connection between practical Medicine and organic and analytical Chemistry. These cursory glances bring vividly to our view the true position and relationships of medical science and practice. It is a very common error, by no means limited to the popular or non-professional mind, that Medicine is an isolated study, made up of cloistered theories and specific formulas, to be learned by rote and applied by routine —that its language, even, is but a mixed jargon of ancient origin, retained only to mystify and mislead the uninitiated. Instead of this, however, we see that Medicine has a legitimate and broad foundation, reaching deeply and securely to the very centre of all the various branches of natural science and philosophy.

Disease, instead of being some mysterious spiritual entity, pervading and disturbing the human body, or some imaginary concoction of humors, is at once recognized as a simple deviation of some organ or structure from its natural condition, induced by some cause or agency, and taking place in accordance with laws known and appreciable, and its progress capable of being measured from day to day. Hence a knowledge of the natural condition and function of each organ and tissue must precede, and constitute the basis of all knowledge of disease. To gain this necessary antecedent knowledge we must enter the wide field of natural history, trace the successive development of different species, orders, and genera, of living beings; that we may recognize more certainly the connection between the development of certain organs and the manifestation of certain functions. We must call to our aid Chemistry, with her fixed laws and most careful manipulations, to analyze the solids and fluids of which each organ is composed, as also the changes which these undergo in the progress of disease; and to complete the task, we must trespass on Natural Philosophy for an instrument, with which to view in their natural position, the relations and qualities of each cell, fibre, and molecule. Nor is this all, or even the half of the field upon which we have entered. We have thus far only found the organ or tissue, and determined its structure, composition, and function. In other words, we have taken the acorn, examined its structure, analyzed its several parts, and recognized in it the elements of the future oak.

But what are the exterior agencies or elements that must be brought to bear on it, to awaken the latent principle of life and cause its rudimentary radicle to extend itself downward and its plumule to shoot upward? So of the organs and tissues, the solids and fluids, of the human body: when we have ascertained their existence, unravelled their structure, and separated their component parts, the next step is, to inquire into the relation each bears to all the others, and to the impression or action of all surrounding objects and influences. And here it is that we come at once to require the most intimate knowledge of the air we breathe, the food we eat, the liquids we drink, with those more occult and mysterious agents, called light, caloric, electricity, &c., involving in their remote ramifications, not only the departments of Botany, Geology, and Meteorology, but the whole range of physical and moral sciences. Not only do all these agents and influences bear an intimate and necessary relation to the human system in a healthy condition, but their perverted and undue action constitute them the chief causes of disease.

Even my non-professional hearers will thus perceive clearly, I trust, that legitimate Medicine, instead of being a mere system of theoretical opinions, emanating from the cloistered conceptions of some erratic genius, is, in truth, but a part, and a most important part too, of the great field of natural science and philosophy, applied to the most noble and beneficent purpose of allting haveiuman suffering and of prolonging human life.

And hence, to talk of *rival* systems of medicine is just as absurd as to talk of rival systems of chemistry, or of mechanics. For so certain as man constitutes a link in the great chain of animate beings, susceptible of being acted on by all the natural agents that surround him with their almost endless variety of combinations, so certain it is, that a knowledge of his diseases involves a more or less intimate knowledge of the adjoining links in the chain, as well as of the exterior agents capable of acting on them.

Therefore, I repeat, that medical science is simply a part of the natural sciences and philosophy of the present day; while medical art is the skilful application of such sciences to the prevention, alleviation, and cure of disease.

For more than two thousand years the two have been linked together by indissoluble bonds. If one has progressed from the most crude jumble of vain, useless, and fanciful speculations, to the form of compact and demonstrative sciences, illuminating and advancing in a wonderful degree almost every human art and employment, so has the other. The same rigid, demonstrative system of investigation, aided by the same instruments and resources, is now being applied to both. And, we may add, with just feelings of pride, that in all ages, the most renowned cultivators of all the sciences have belonged to the ranks of our profession. This, however, results legitimately and necessarily from the fact, that a proper study of man in health and disease, is but the climax or crowning point, reached with facility, only, through a study of all the rest. It is true that medicine has its crude and fanciful offshoots, called *isms* and *pathys*, which cling to it like, here and there, a withered branch to the green and sturdy oak. And the same is equally true of every other branch of science. Thus, if Hanneman drew so largely upon a fertile imagination as to persuade himself that almost all diseases originate from the *itch*, and are to be cured only on the single fanciful principle of *similia similibus curantur;* so did Metcalfe give to the admirers of general science two ponderous *tomes*, to prove that caloric is the great and only motor power of the universe, causing alike the sublime movements of the spheres, the minutest oscillations of the microscopic animalculæ, and constituting even the principle of life itself. If Preissnitz has astonished the fashionable world with the pretended virtues of cold water, so have the necromancing performers on mesmerism, psychology, and biology, bewildered and confounded learned professors of theology, philosophy, and natural sciences. And even the most selfish and secular employments of life are not free from equally fanciful delusions, as is well illustrated by the fact that a prominent moneyed institution in the midst of this enlightened city, has seriously attempted to conduct its daily business in strict accordance with pretended revelations from the "spirit" world, with silly women for mediums of communication. But if the insane "spiritual" system of banking developed in our midst, has left the fixed and well known principles of exchange and laws of trade undisturbed; if the sublime but visionary speculations of Metcalfe have left the laws of gravitation and the movements of the universe as Newton found them; so have hydropathy and homœopathy failed to remove one stone from the broad foundations of medical science. The truth is, that quackery exists in every department of science and industry, and is the legitimate and necessary result of systems and modes of education, that develope unequally and irregularly the higher faculties of the human mind. Such unequal and irregular mental developments will ever produce erratic geniuses, who will bear the same relation to the great mass of cultivated mind, that the meteors and shooting stars do to the rest of the planetary system. They may *dazzle* by their brilliancy, or throw a gleam of fitful light across the mental heavens, as they rush madly from their appropriate spheres, but they leave only thicker darkness behind.

Such, gentlemen, are my views of the nature and relations of the broad field upon which you have now entered. Let us next inquire, whether its honorable cultivation is attended by difficulties or dangers; whether its broad surface is one vast and sunny plain, on which gems and flowers in endless variety and beauty greet us at every step; or whether these latter are only seen here and there peeping out amidst such rugged rocks, towering cliffs, and dark ravines, as to make the toil and danger incident to their possession outweigh the value of the treasure when obtained. Alas! I fear the latter has been, and will be, the conclusion arrived at by too large a proportion of our professional brethren.

The first difficulty that meets the physician as he steps over the threshold of the profession, consists in the very extent of the field to be occupied, and the protracted period of labor required for its cultivation. Hence, many, after proceeding just far enough to gain the summit of some minor eminence, where they can gain a partial view of the surrounding landscape, shrink from the further prosecution of their task, and either content themselves with their present position, or turn aside to employments less laborious, and more productive of mere pecuniary gain.

The second difficulty consists in a want of preparation on the part of the individual essaying to study and practice the healing art. Not a few find themselves within the pale of our profession, with so defective a preliminary education that they are wholly incapable of perceiving either the extent of the field upon which they have entered, or the materials and conditions necessary for its successful cultivation. To such, the relations of man to the almost endless variety of agents capable of acting upon him, and to the vast chain of animated nature, are veiled in impenetrable obscurity. Such are like the husbandmen of some barbarous countries, who are destitute of the very implements necessary for the successful prosecution of their labor; and are hence required to delve for years with a rude spade, on a spot of ground which the enlightened agriculturist, with his modern plough and harrow, would cultivate much more effectually in a single day.

A third difficulty arises from that inordinate thirst for pecuniary emolument, which so often gains the ascendancy over every purer and nobler impulse of the mind, and effectually prevents that earnest and unceasing devotion to study which is necessary to command success. When this difficulty is connected with that unequal or erratic development of the mental faculties, to which I have already alluded, it almost inevitably leads to some one of those vulgar and dishonest artifices or tricks to attract attention and increase the pecuniary resources, which have so often disgraced members of our profession, and not unfrequently seduced them into the adoption of whatever *pathy* or *ism* seemed most popular at the time.

And still another difficulty is found in the great amount of ignorance that exists in every circle of human society, in regard to everything relating to health and life; and consequently, the frequent failure to appreciate, in any adequate degree, those exhibitions of genuine skill and high attainment, that have cost the exhibitors years of the most careful and profound research. Indeed, there can be but few things more seriously disheartening to a cultivated and ambitious mind, than to find his most anxious and faithful attentions regarded as scarcely more valuable than those of the boastful and ignorant pretender, who often treads with bold and reckless step where even angels would enter with fear and trembling.

I must hasten, however, from this branch of my subject, lest you gain the impression that the professional field before you is little better than a barren desert covered with rocks and brambles, without a single gem to enrich, or a flower to beautify its surface. Notwithstanding all the difficulties to which we have alluded, and many more that actually exist, the science and practice of medicine presents, at once, the most fertile, varied, and interesting field of labor open to the possession of man. To the lovers of pure science, its departments of descriptive and microscopic anatomy, physiology, and medical botany, furnish subjects of most thrilling interest and beauty. Within their limits are to be found the most gorgeous flowers that adorn the great garden of Nature; the most wonderful views of the intricate structures, wholly invisible to the unassisted eye; the most ingenious combinations of mechanical powers, constituting the most complex mechanisms, and yet working with all the harmony that characterizes the simplest operations of Nature. To the lovers of the more speculative and philosophical themes of study, the same branches present the *mind* with all its varied and sublime powers, its almost boundless capacity for expansion and improvement, and its mysterious union with matter in so close a relation that *thought* is made to beam in the eye, linger on the tongue, and simple *emotion* to thrill, like the lightning's flash, even the remotest microscopic fibre of the material frame. To the lovers of the practical and useful, the utilitarians of the present day—even to that class who stoically meet every proposition with the exclamation, *Cui bono?* What good?—the practical application of all the knowledge that can be gleaned from the whole vast range of human sciences, to the most desirable and useful of all purposes, namely, the maintenance of health and the cure of disease, cannot excite any other feelings than those of intense interest. To the philanthropist, the genuine lover of God and man, the access which our profession gives to the hearthstones and the hearts of the poor, the unfortunate and the erring of our race, and the ample and varied means it furnishes for soothing alike the pains of the body and the agonies of the mind, makes it, at least, equal in importance to any other earthly pursuit. While to the true statesman and legislator, whose aim is to promote the civilization, advancement, and happiness of man, more than the gratification of a personal and selfish ambition, the study of our profession can alone furnish that knowledge of the causes of disease, which will enable him to adopt such measures as will judiciously regulate the sanitary condition of towns and cities, promote the public health, and preserve the lives of citizens.

Having thus surveyed, rapidly and hastily, it is true, the ample field upon which you have entered—pointed out its nature, relations, difficulties, and objects—it only remains for me to ask you individually and collectively, whether you are prepared to prosecute its cultivation with that persevering industry and high resolve, that will result in an abundant harvest of honor to yourselves, honor to your Alma Mater, and justice to the communities in which you may live? If any of you have chosen the profession of medicine merely as an honorable mode of gaining a livelihood, or for the pecuniary emoluments which its practice will afford, I advise you candidly to lose no time in retracing your steps, and choosing some other pursuit. Not, however, from any sentimental idea that the practice of medicine is exclusively a *benevolent* pursuit, or that its devotees should be regardless of all pecuniary considerations. No. The maxim, that "the laborer is worthy of his hire," is as applicable to the physician as to any other member of society. It is true, that he, perhaps more than any others, should be kind to the poor, and lenient to the unfortunate; but of those who are able, he should be as rigid in requiring his pay as other men, and for this if for no other reason, that he may be the better able to be kind to such as have none of this world's goods. I advise you thus, however, because even under the most favorable circumstances, the pecuniary emoluments of the physician bear no adequate proportion to his labors by day and by night, his exposures in the midst of pestilence and death, and the vast responsibilities imposed on him. In view of these things, the men who dig our canals and ditch our prairies, are far better remunerated than the most renowned physician in our prosperous and comparatively happy country.

But, gentlemen, if you have chosen this profession from an ardent love for those branches of science that constitute its broad and ample foundation, or from a pure and noble desire to spend life in applying the knowledge derived from the study of these sciences to the holy purpose of alleviating the sufferings of your fellow-men—or from both these motives combined—then I most cheerfully bid you God-speed, and again welcome you as co-laborers in this our own chosen vineyard. For, if excited to action by such motives, I should feel confident that you will not only gain for yourselves a pecuniary competence, which is all that we can enjoy, but your career will be such that you will leave the world better for your having lived in it.

One thing more, gentlemen, and I have done. When the business-man of honor procures the signature of his neighbor to enhance his own credit, or afford additional security to the capitalist, he feels under the strongest possible obligations to return that signature uninjured to its generous owner. I see in the hands of each one of you, a scroll, and on that scroll you have the names of my colleagues and myself. You have procured them as your professional endorsers, emphatically to give you additional credit with the world, and are hence under the most sacred obligations to maintain that credit, and consequently the names linked with it, unsullied by a single stain up to the very end of life. If, in the midst of the toils and cares, and, it may be, the misfortunes and even poverty that await you in life, the vicious heirs of worldly wealth should hold out to you their tempting bribes, or the numerous dishonorable artifices sometimes practised with temporary success by others, should suggest themselves, remember that your conduct involves not your own honor only, but also that of your professional endorsers, and of all your professional brethren; and, remembering these things, turn from the temptation as you would from the venomous reptile. The physician, more than any other member of society, is admitted not only to the family fire-side and the heart, but often also to the most confidential relations of individuals and families. For this reason, if for no other, his own life should be an example of such spotless purity, as to stand a perpetual rebuke to the vicious, and an equally perpetual encouragement to those who do well.

If, in giving heed to these precepts, you should occasionally see the unprincipled and vicious spring up, and, for a time, "flourish like the green bay-tree," be not discouraged or envious, but wait patiently, and in a little time you shall seek for them and they will not be found. In a word, ever keep in view the great truth, that, temporally speaking,

"It is not all of Life to live,
Nor all of death to die."

The period allotted to men here, is, indeed, brief; and its days flee like the mists of the morning. But there is a life, the days of which are not numbered; and there is an existence where every virtue shall receive its full reward. Hence it is only the dictate of common prudence and reflection, that the true end of all our toil here is to secure the bliss of an endless life hereafter. Therefore, there is no fanaticism in the remark, that it is infinitely better to be followed to the grave by weeping widows and orphans, who shall exclaim, as they turn from the humble mound, that a *good* man has fallen, than to have our last resting-place marked by a towering and gilded monument, erected from the wages of iniquity With these admonitions, young gentlemen, we bid you go, with an earnest prayer that you may be a blessing to yourselves and your race.

The whole wide world is before you; and whatever spot you may select as your own particular field, remember to cultivate the kindest feelings of friendship and sympathy for your brethren engaged in the same arduous calling.

Remember, too, that sentiment so beautifully expressed in our national code of Medical Ethics, that, "on emergencies for which no professional man should be unprepared, *a steady hand, an acute eye, and an unclouded head* may be essential to the wel being, and even to the life, of a fellow creature.' —*Farewell.*

